# Stress Resilience and Risk of Psychiatric Disorders After Childhood Bereavement

**DOI:** 10.1001/jamanetworkopen.2025.19706

**Published:** 2025-07-09

**Authors:** Ludvig D. Bjørndal, Yufeng Chen, Donghao Lu, Isabell Brikell, Ralf Kuja-Halkola, Brian M. D’Onofrio, Andreas H. Lundin, Paul Lichtenstein, Henrik Larsson, Hilda Björk Daníelsdóttir, Katja Fall, Unnur A. Valdimarsdóttir, Fang Fang

**Affiliations:** 1 PROMENTA Research Center, University of Oslo, Oslo, Norway; 2 Unit of Integrative Epidemiology, Institute of Environmental Medicine, Karolinska Institutet, Stockholm, Sweden; 3 Department of Medical Epidemiology and Biostatistics, Karolinska Institutet, Stockholm, Sweden; 4 Department of Global Public Health and Primary Care, University of Bergen, Bergen, Norway; 5 Department of Biomedicine, Aarhus University, Aarhus, Denmark; 6 Department of Psychological and Brain Sciences, Indiana University, Bloomington; 7 Swedish Defence Conscription and Assessment Agency, Ministry of Defence, Stockholm, Sweden; 8 Department of Neuroscience, Karolinska Institutet, Stockholm, Sweden; 9 School of Medical Sciences, Örebro University, Örebro, Sweden; 10 Centre of Public Health Sciences, Faculty of Medicine, University of Iceland, Reykjavik, Iceland

## Abstract

**Importance:**

Childhood bereavement increases the risk of common psychiatric disorders later in life. However, the role of stress resilience in this association remains underexplored.

**Objective:**

To assess whether stress resilience mediates the association between childhood bereavement and psychiatric disorder risk in adulthood.

**Design, Setting, and Participants:**

Three matched cohort studies were performed using data recorded in the Swedish Military Conscription Register. Individuals with childhood loss of either a parent or a sibling (19 162 participants), a parent (16 247 participants), or a sibling (3023 participants) due to death from 1987 to 2020, together with 10 unexposed individuals per exposed individual, were matched on sex, birth year, and county of birth. All participants had available stress resilience measure at conscription. Data were analyzed from February to December 2024.

**Exposures:**

Childhood bereavement was ascertained from the Swedish Multi-Generation and Causes of Death Registers.

**Main outcomes and Measures:**

Incident diagnosis of depression, anxiety, substance use disorder, and stress-related disorder was ascertained from the Swedish Patient Register. Cox models were used to estimate the association between childhood bereavement and risk of postconscription psychiatric disorders after multivariable adjustment. Causal mediation analysis was conducted to examine if stress resilience measured at conscription mediated this association.

**Results:**

Among 1 733 085 conscripted individuals (median [IQR] age at conscription 18.2 [18.0-18.5] years; 1 707 960 [98.5%] male), the median (IQR) age of individuals exposed to loss of either a parent or a sibling, parent, or sibling was 13.4 (9.6-15.8), 13.7 (10.4-15.9), and 10.7 (5.5-14.9) years, respectively. The crude incidence rate of any psychiatric disorder was 7.9, 8.1, and 6.6 per 1000 person-years among the 3 groups (5.3, 5.8, and 5.5 per 1000 person-years among the respective unexposed groups). A positive association was noted for loss of either a parent or a sibling (HRs ranged from 1.13; 95% CI, 1.06-1.20 for anxiety to 1.31; 95% CI, 1.23-1.40 for substance abuse disorder) and loss of a parent (HRs ranged from 1.10; 95% CI, 1.01-1.20 for stress-related disorders to 1.19; 95% CI, 1.12-1.27 for depression). For loss of a sibling, the statistically significant associations were for any common psychiatric disorder (HR, 1.12; 95% CI, 1.00-1.25) and stress-related disorders (1.27; 95% CI, 1.04-1.55). Stress resilience partially mediated the associations (proportions for loss of either a parent or a sibling ranged from 10.6%-19.4%, for a parent ranged from 15.6%-21.7%, and for a sibling ranged from 6.2% for stress-related disorders to 18.4% for any common psychiatric disorder).

**Conclusions and Relevance:**

In this cohort study of a nationwide Swedish sample, altered stress resilience was found to be one mechanism through which childhood bereavement is associated with risk of psychiatric disorders later in life.

## Key Points

**Question:**

How does stress resilience affect the association between childhood bereavement and psychiatric disorder risk?

**Findings:**

In this cohort study of 1 733 085 individuals, childhood bereavement was associated with increased risk of psychiatric disorders, and this association was partly mediated by stress resilience.

**Meaning:**

These findings suggest that altered stress resilience may be a mechanism through which loss of a parent or sibling in childhood is associated with risk of psychiatric disorders in adulthood.

## Teaser

This cohort study evaluates whether stress resilience mediates the association between childhood bereavement and psychiatric disorder risk in adulthood.

## Introduction

Bereavement increases the risk of common psychiatric disorders, such as depression, anxiety, and substance use.^1^ Specifically, it has been found that children who experience the death of a parent have an increased risk of psychiatric disorder diagnoses and symptoms^2,3,4,5,6^ and hospitalizations.^7,8^ Similarly, sibling loss has been associated with elevated risk of mental disorders and symptomatology.^9,10^ Losing a parent or sibling is not uncommon; 1 US study recently estimated that 7% of children will experience the death of a parent or sibling by age 18.^11^ Consequently, childhood bereavement constitutes an important risk factor for mental health problems later in life.

Recent research has examined the role of resilience in how stressful experiences like bereavement are related to the risk of psychiatric disorders. While a minority of bereaved individuals experience prolonged psychological disruption, many maintain healthy psychological functioning after such events.^12,13,14^ Resilience is conceptualized differently across studies, including as a stable pattern of healthy functioning following a stressful event like bereavement (eg, defined as the absence of mental disorder after a stressor) or as an individual trait or characteristic measured using self-report methods.^15,16,17^ Higher trait resilience has broadly been associated with lower risk of depressive and anxiety symptoms^18,19,20,21^ and lower risk of adverse physical health outcomes across life, such as heart disease,^22^ stroke,^23^ and hypertension.^24^

The effects of bereavement exposure on risk of psychiatric disorders may vary between individuals with different resilience and coping abilities.^13^ Moreover, bereavement in early childhood has been associated with lower stress resilience measured in late adolescence.^25^ As such, childhood bereavement may in part influence the risk of psychiatric disorders through altering stress resilience, which in turn affects the risk of psychiatric disorders across life (ie, a mediating effect of stress resilience). One recent meta-analysis found that trait resilience partly mediated the association between childhood stressful events and depressive symptoms in adulthood.^21^

Few studies have comprehensively assessed the role of stress resilience in potentially mediating the effect of bereavement exposure on risk of psychiatric disorders. The majority of existing studies on resilience and mental health have methodological shortcomings, such as retrospective reporting of life stressors which may be affected by reporting bias^21^ and small and nonrepresentative samples.^20^ Crucially, existing meta-analyses are solely based on cross-sectional data,^18,21^ highlighting the need for longitudinal studies with prospectively and independently collected information on bereavement and mental health. In addition, most existing studies of childhood bereavement and psychiatric disorder risk have been limited to short follow-up duration (eg, the first 2 years after bereavement).^3,5^ As such, long-term effects of childhood bereavement on psychiatric disorder risk are not well understood.

To this end, we leveraged population-based Swedish registers to prospectively assess associations between bereavement exposures and risk of common psychiatric disorders in adulthood, with a focus on deciphering the potential mediating role of stress resilience. We hypothesized that stress resilience partially mediates the association of childhood bereavement with the risk of psychiatric disorders.

## Methods

This study was approved by the Swedish Ethical Review Authority. Informed consent is not required for pseudoanonymized register-based research according to Swedish law. This study is reported according to Strengthening the Reporting of Observational Studies in Epidemiology (STROBE) reporting guideline.^26^

### Study Design

The population for this study included all Swedish men and women with available data on stress resilience in the Swedish Military Conscription Register (SMCR)^27^ from 1969 to 2020. SMCR includes data on Swedish individuals who underwent assessments for military enlistment at around age 18 years (ie, conscripts). Conscripts were linked to the Swedish Patient Register to identify recorded diagnoses of psychiatric disorders, using the individually unique personal identity numbers assigned to all Swedish residents at the time of birth or immigration.^28^ The Swedish Patient Register includes data on hospital admissions for psychiatric disorders from 1973 (nationwide coverage since 1987) and outpatient specialist care of psychiatric disorders from 2001 (>80% coverage) in Sweden.^29^ Parents and siblings of these conscripts were identified through the Swedish Multi-Generation Register, which includes largely complete data on familial links for individuals born since 1932 in Sweden.^30^ These relatives were further linked to the Causes of Death Register, which contains data on all deaths in Sweden since 1952,^31^ to assess experiences of bereavement due to death of a relative among the conscripts. We defined childhood bereavement as a loss of a parent or sibling during childhood (aged <18 years) due to death.

In the present study, we included conscripts who resided in Sweden in 1987 or later and had available data on stress resilience in the SMCR (eFigure in Supplement 1). We subsequently identified individuals who had experienced childhood bereavement due to loss of a parent, sibling, or either a parent or a sibling. The latter group was constructed to maximize statistical power in analyses. Date of exposure was used as the index date for the exposed individuals. We constructed 3 population-based matched cohorts for analyses of each bereavement type. For each exposed individual, we randomly selected 10 individuals from the study population who had not experienced childhood bereavement before the index date. The index date of the exposed individual was used as the index date for their matched unexposed individuals. As we were interested in incident psychiatric disorders after conscription (ie, in adulthood), exposed and unexposed individuals with a diagnosis of psychiatric disorder before the index date or conscription were excluded. Exposed and unexposed individuals were individually matched by sex, birth year, and birthplace (county of birth). Unexposed individuals were further required to have the specific type of relative (ie, sibling and/or parent) to be eligible as an unexposed person.

Within each cohort, the exposed and unexposed individuals were followed up from the date of conscription until the first diagnosis of the studied psychiatric disorders, death, emigration, first exposure to childhood bereavement (for unexposed individuals from when they contributed as exposed individual), or the end of follow-up (December 31, 2020), whichever occurred first.

### Ascertainment of Psychiatric Disorders

We identified incident psychiatric disorders from the Swedish Patient Register, using both the primary and secondary diagnoses of either an inpatient or outpatient hospital visit, based on Swedish revisions of the *International Classification of Diseases, Ninth Revision* (*ICD-9*; years 1987-1996), and *Tenth Revision* (*ICD-10*; from 1997 onwards), codes. We included depression, anxiety, substance use disorder, and stress-related disorders (individually and combined as common psychiatric disorders) as outcomes. The diagnostic codes used in the study are reported in eTable 1 in Supplement 1.

### Measurement of Stress Resilience

The SMCR includes data on assessments of stress resilience.^24,25,27^ Stress resilience was measured based on an interview conducted by a trained psychologist, which was aimed to assess coping ability among conscripts with regards to various aspects of military service. The interview included assessments of previous conflicts, adjustment problems, and emotional stability. Stress resilience was assessed using a stanine scale (a 9-point scale with a normal distribution and a standard deviation of 2) and this score was dichotomized as low (1-3) and high (4-9). Previous research on stress resilience within SMCR has referenced studies indicating that the stress resilience measure is validated as a predictor of conscription performance and has high interrater reliability.^25^

### Covariates

We obtained information on the educational level of the parents and family income of the study participants 1 year before the index date from the Swedish Longitudinal Integration Database for Health Insurance and Labor Market. Family history of psychiatric disorders, defined as having any diagnosed psychiatric disorder among biological parents or full siblings before the index date, was ascertained from the Swedish Patient Register.

We also included measures of physical fitness and cognitive ability, as these have been shown to be related to stress resilience in SMCR^25^ and psychopathology.^32^ Physical fitness was assessed using a bicycle ergometer and on a stanine scale and was categorized as low (0-4) or high (5-9). Cognitive ability was assessed using tests of verbal, spatial, logical inductive, and technical abilities and summarized on a stanine score.^27^ The cognitive ability score was categorized as low (1-3) or high (4-9).

### Statistical Analysis

We conducted statistical analyses in 3 broad steps. First, we described characteristics of the study participants and estimated the overall association between childhood bereavement and risk of psychiatric disorders. Second, we examined if childhood bereavement was associated with stress resilience in late adolescence, and if stress resilience in late adolescence was associated with risk of psychiatric disorder later in life. Third, we assessed if stress resilience mediated the effect of childhood bereavement on risk of psychiatric disorders in adulthood.

For the first set of analyses, we initially described the characteristics of the study participants by bereavement type, including age, sex, parental educational level, annual family income level, family history of psychiatric disorders, year of conscription; and stress resilience, physical fitness, and cognitive ability measured at conscription. Second, we calculated the crude incidence rates by dividing the number of cases with incident psychiatric disorders by accumulated person-years at risk for individuals who were exposed or unexposed to the different types of bereavement. Third, we calculated mean hazard ratios (HRs) using stratified Cox models with time since the date of conscription as the underlying timescale, conditioned on matching identifiers (birth year, sex, and birthplace) and adjusted for the year of conscription (model 1). Model 2 was additionally adjusted for parental educational level, family income, and family history of psychiatric disorders. Model 3 was additionally adjusted for physical fitness and cognitive ability at conscription. The proportional hazards assumption was tested using Schoenfeld residuals^33^ with no violation detected. We conducted subgroup analyses by sex, age at index date (in years: <6, 6-12, and >12-18), family income, family history of psychiatric disorders, calendar year of conscription (1987-2006 or 2007-2020), physical fitness, cognitive ability, and sex of the deceased parent or sibling. Likelihood ratio tests were conducted to assess heterogeneity of estimates across subgroups.

In the main analysis, we excluded participants who were diagnosed with a psychiatric disorder before the index date or conscription as we were primarily interested in psychiatric disorders diagnosed in adulthood. To examine the soundness of study results to such exclusion, we performed a sensitivity analysis also including participants who were diagnosed with a psychiatric disorder before conscription and separately assessed the associations of bereavement with the risk of psychiatric disorders before and after conscription. In this analysis, time since the index date was used as the underlying time scale

The second set of analyses was based on the 3 sets of matched cohorts previously constructed. We first examined the association of childhood bereavement with stress resilience measured in late adolescence using logistic regression models (ie, childhood bereavement as exposure and stress resilience at conscription as outcome). We subsequently examined the association between stress resilience measured at conscription and risk of psychiatric disorders later in life using Cox models with time since the date of conscription as the underlying time scale (ie, stress resilience as the exposure and psychiatric disorders as outcome). Three models were run: model 1 adjusted for sex, birthplace, birth year, and conscription year; model 2 additionally adjusted for parental educational level, family income, and family history of psychiatric disorders; and model 3 additionally adjusted for physical fitness and cognitive ability at conscription.

For the third set of analyses, we conducted causal mediation analyses to examine if the association between childhood bereavement and risk of psychiatric disorders later in life was mediated by stress resilience, using the SAS macro %mediation developed by Valeri and VanderWeele.^34^ We quantified natural direct effects (ie, the effect of bereavement while stress resilience is held constant), natural indirect effects (ie, the effect of bereavement that operates through stress resilience), total effect (including both natural direct and indirect effects), and the proportion of effects mediated by stress resilience.^35,36^

All analyses were conducted in SAS statistical software version 9.4 (SAS Institute) and R version 4.3.3 (R Project for Statistical Computing) from February to December 2024. A 2-sided *P* < .05 was considered statistically significant.

## Results

### Descriptive Statistics and Associations Between Childhood Bereavement and Psychiatric Disorders

Among 1 733 085 individuals included in the study, the median (IQR) age at conscription was 18.2 (18.0-18.5) years and 1 707 960 participants (98.5%) were male. A total of 16 247 individuals experienced childhood bereavement due to loss of a parent, 3023 due to loss of a sibling, and 19 162 due to loss of either a parent or a sibling. The median (IQR) age at exposure to bereavement due to loss of either a parent or a sibling was 13.4 (9.6-15.8), due to loss of a parent was 13.7 (10.4-15.9), and due to loss of a sibling was 10.7 (5.5-14.9) years (Table 1). During a median follow-up of 18.4 (12.5-24.2), 18.7 (12.5-24.3), and 17.2 (12.3-22.6) years, the crude incidence rates of the studied common psychiatric disorders were 7.9, 8.1, and 6.6 per 1000 person-years among individuals who had experienced loss of either a parent or sibling, parent, and sibling, respectively (5.3, 5.8, and 5.5 for their corresponding unexposed groups).

**Table 1.** Characteristics of Study Cohorts for Exposure to Childhood Bereavement

Characteristics	Patients, No. (%)					
	Loss of either parent or sibling		Loss of parent		Loss of sibling	
	Exposed (n = 19 162)	Unexposed (n = 191 437)	Exposed (n = 16 247)	Unexposed (n = 162 375)	Exposed (n = 3023)	Unexposed (n = 30 205)
Age at index date, median (IQR), y^a^	13.4 (9.6-15.8)	13.4 (9.6-15.8)	13.7 (10.4-15.9)	13.7 (10.3-15.9)	10.7 (5.5-14.9)	10.7 (5.5-14.9)
Follow-up, median (IQR), y	18.4 (12.5-24.2)	19.0 (13.1-24.6)	18.7 (12.5-24.3)	19.2 (13.1-24.7)	17.2 (12.3-22.6)	17.4 (12.6-22.9)
Sex						
Male	18 567 (96.9)	185 660 (97.0)	15 767 (97.0)	157 665 (97.1)	2906 (96.1)	29 060 (96.2)
Female	595 (3.1)	5777 (3.0)	480 (3.0)	4710 (2.9)	117 (3.9)	1145 (3.8)
Parental educational level, y						
<9	2160 (11.3)	5249 (2.7)	2035 (12.5)	4810 (3.0)	139 (4.6)	744 (2.5)
9-12	11 397 (59.5)	99 623 (52)	9766 (60.1)	86 042 (53.0)	1696 (56.1)	15 869 (52.5)
>12	5306 (27.7)	85 252 (44.5)	4181 (25.7)	69 211 (42.6)	1153 (38.1)	13 360 (44.2)
Unknown	299 (1.6)	1313 (0.7)	265 (1.6)	2312 (1.4)	35 (1.2)	232 (0.8)
Annual family income level						
Lowest 20%	1116 (5.8)	1593 (0.8)	1088 (6.7)	1402 (0.9)	34 (1.1)	240 (0.8)
Middle	14 540 (75.9)	108 305 (56.6)	12 599 (77.5)	92 879 (57.2)	2022 (66.9)	18 604 (61.6)
Top 20%	3291 (17.2)	80 952 (42.3)	2356 (14.5)	66 404 (40.9)	955 (31.6)	11 258 (37.3)
Unknown	215 (1.1)	587 (0.3)	204 (1.3)	1690 (1.0)	12 (0.4)	103 (0.3)
Family history of psychiatric disorders						
No	14 257 (74.4)	178 595 (93.3)	11 721 (72.1)	148 218 (91.3)	2604 (86.1)	28 430 (94.1)
Yes	4862 (25.4)	12 833 (6.7)	4483 (27.6)	13 195 (8.1)	419 (13.9)	1775 (5.9)
Unknown	43 (0.2)	9 (0.1)	43 (0.3)	962 (0.6)	0	0
Year of conscription						
1987-2006	16 246 (84.8)	162 605 (84.9)	13 893 (85.5)	138 934 (85.6)	2433 (80.5)	24 391 (80.8)
2007-2020	2916 (15.2)	28 832 (15.1)	2354 (14.5)	23 441 (14.4)	590 (19.5)	5814 (19.2)
Stress resilience at conscription^b^						
High (4-9)	13 289 (69.4)	151 151 (79.0)	11 138 (68.6)	126 505 (77.9)	2219 (73.4)	23 683 (78.4)
Low (1-3)	5873 (30.6)	40 286 (21.0)	5109 (31.4)	35 870 (22.1)	804 (26.6)	6522 (21.6)
Physical fitness at conscription^c^						
High (5-9)	13 521 (70.6)	148 990 (77.8)	11 427 (70.3)	125 492 (77.3)	2160 (71.5)	22 835 (75.6)
Low (0-4)	2670 (13.9)	21 567 (11.3)	2288 (14.1)	19 104 (11.8)	396 (13.1)	3413 (11.3)
Unknown	2971 (15.5)	20 880 (10.9)	2532 (15.6)	17 779 (10.9)	467 (15.4)	3957 (13.1)
Cognitive ability at conscription^d^						
High (4-9)	14 878 (77.6)	160 165 (83.7)	12 542 (77.2)	134 315 (82.7)	2422 (80.1)	25 368 (84.0)
Low (1-3)	3726 (19.4)	28 108 (14.7)	3223 (19.8)	25 317 (15.6)	522 (17.3)	4292 (14.2)
Unknown	558 (2.9)	3164 (1.7)	482 (3.0)	2743 (1.7)	79 (2.6)	545 (1.8)

^a^ Date of bereavement for the exposed participants and matching date for their sex, birth year, and birthplace individually matched unexposed participants.

^b^ Stress resilience was measured based on an interview conducted by a trained psychologist.

^c^ Physical fitness was assessed using a bicycle ergometer and on a stanine scale.

^d^ Cognitive ability was assessed using tests of verbal, spatial, logic inductive, and technical abilities and summarized on a stanine scale.

Among participants exposed to loss of a parent, a smaller proportion had high parental educational levels and family income in the top 20%, while a substantially higher proportion had a family history of psychiatric disorders (Table 1). More individuals who had experienced loss of a sibling had a family history of psychiatric disorders.

Positive associations were noted between loss of either a parent or sibling in childhood and common psychiatric disorder combined (HR, 1.21; 95% CI, 1.16-1.26), depression (HR, 1.21; 95% CI, 1.13-1.29), anxiety (HR,1.13; 95% CI, 1.06-1.20), substance use disorder (1.31; 95% CI, 1.23-1.40), and stress-related disorders (HR, 1.19; 1.09-1.29) (Table 2), and for loss of a parent (common psychiatric disorders HR, 1.14; 95% CI, 1.09-1.19; depression HR, 1.19; 95% CI 1.12-1.27; anxiety HR, 1.11; 95% CI, 1.04-1.18; substance abuse disorder HR, 1.15; 95% CI, 1.07-1.22; stress-related disorders HR, 1.10; 95% CI, 1.01-1.20). For loss of a sibling, the only statistically significant associations were noted for any common psychiatric disorder (HR, 1.12; 95% CI, 1.00-1.25) and stress-related disorders (HR, 1.27; 95% CI, 1.04-1.55). We found no statistically significant heterogeneity in the subgroup analyses by sex, age at index date, family income, family history of psychiatric disorders, year of conscription, physical fitness, cognitive ability, or sex of the deceased parent or sibling (eTable 2 in Supplement 1). The sensitivity analysis including participants with a psychiatric disorder diagnosed before conscription showed largely similar results as for adult psychiatric disorders, although the associations were greater for childhood psychiatric disorders (eTable 3 in Supplement 1).

**Table 2.** Crude IRs and HRs With 95% CIs for Risks of Adult Psychiatric Disorders Among Individuals Exposed to Childhood Bereavement Compared With Matched Unexposed Individuals

Outcomes	No. of cases (IR) during follow-up^a^		HR (95% CI)^b^		
	Exposed individuals	Unexposed individuals	Model 1^c^	Model 2^d^	Model 3^e^
Exposed to loss of either parent or sibling					
Common psychiatric disorders	3055 (7.9)	20 984 (5.3)	1.48 (1.43-1.54)	1.24 (1.19-1.29)	1.21 (1.16-1.26)
Depression	1334 (3.2)	8841 (2.2)	1.50 (1.42-1.59)	1.25 (1.17-1.33)	1.21 (1.13-1.29)
Anxiety	1414 (3.4)	9733 (2.4)	1.44 (1.36-1.52)	1.17 (1.10-1.24)	1.13 (1.06-1.20)
Substance abuse disorder	1392 (3.4)	8134 (2.0)	1.73 (1.63-1.83)	1.35 (1.27-1.44)	1.31 (1.23-1.40)
Stress-related disorders	831 (2.0)	5427 (1.3)	1.53 (1.42-1.64)	1.21 (1.12-1.32)	1.19 (1.09-1.29)
Exposed to loss of parent					
Common psychiatric disorders	2693 (8.1)	19 505 (5.8)	1.41 (1.35-1.47)	1.16 (1.11-1.21)	1.14 (1.09-1.19)
Depression	1185 (3.4)	8211 (2.3)	1.45 (1.36-1.54)	1.22 (1.14-1.30)	1.19 (1.12-1.27)
Anxiety	1252 (3.6)	9003 (2.6)	1.40 (1.32-1.49)	1.14 (1.07-1.22)	1.11 (1.04-1.18)
Substance abuse disorder	1249 (3.6)	8181 (2.3)	1.52 (1.43-1.62)	1.16 (1.09-1.24)	1.15 (1.07-1.22)
Stress-related disorders	718 (2.0)	5171 (1.5)	1.39 (1.29-1.51)	1.11 (1.02-1.21)	1.10 (1.01-1.20)
Exposed to loss of sibling					
Common psychiatric disorders	388 (6.6)	3287 (5.5)	1.20 (1.08-1.33)	1.13 (1.01-1.26)	1.12 (1.00-1.25)
Depression	164 (2.7)	1396 (2.3)	1.20 (1.02-1.41)	1.11 (0.94-1.31)	1.10 (0.93-1.30)
Anxiety	177 (2.9)	1507 (2.4)	1.19 (1.01-1.39)	1.09 (0.93-1.28)	1.09 (0.93-1.28)
Substance abuse disorder	148 (2.4)	1301 (2.1)	1.13 (0.95-1.35)	1.06 (0.89-1.27)	1.06 (0.89-1.27)
Stress-related disorders	120 (1.9)	858 (1.4)	1.41 (1.16-1.72)	1.30 (1.07-1.59)	1.27 (1.04-1.55)

Abbreviations: HR, hazard ratio; IR, incidence rate.

^a^ Incidence rate of psychiatric disorders per 1000 person-years.

^b^ Estimates were calculated from Cox models with time since the conscription date as the underlying time scale.

^c^ Model 1 was stratified by matching identifier (sex, birth year, and birthplace), and adjusted for conscription year.

^d^ Model 2 was additionally adjusted for parental educational level, family income, and family history of psychiatric disorders.

^e^ Model 3 was further adjusted for physical fitness and cognitive ability at conscription.

### Associations Between Childhood Bereavement, Stress Resilience, and Risk of Psychiatric Disorders

Childhood bereavement due to loss of either a parent or sibling (OR, 1.22; 95% CI, 1.17-1.27), parent (OR, 1.17; 95% CI, 1.12-1.23), or sibling (OR, 1.13; 95% CI, 1.02-1.27) was associated with a higher probability of having low stress resilience at conscription (Table 3). Low stress resilience was associated with increased risk of all studied common psychiatric disorders later in life (HRs ranged from 1.59; 95% CI, 1.34-1.88 for stress-related disorders to 2.09; 95% CI, 1.83-2.39 for depression) (Table 4).

**Table 3.** Association Between Childhood Bereavement and Stress Resilience in Late Adolescence

Exposed to childhood bereavement	Stress resilience, No. (%)		OR (95% CI) for low stress resilience^a^		
	High	Low	Model 1^b^	Model 2^c^	Model 3^d^
Loss of either parent or sibling					
No	151 151 (91.9)	40 286 (87.3)	1 [Reference]	1 [Reference]	1 [Reference]
Yes	13 289 (8.1)	5873 (12.7)	1.67 (1.61-1.72)	1.30 (1.26-1.35)	1.22 (1.17-1.27)
Loss of parent					
No	126 505 (91.9)	35 870 (87.5)	1 [Reference]	1 [Reference]	1 [Reference]
Yes	11 138 (8.1)	5109 (12.5)	1.62 (1.57-1.68)	1.24 (1.20-1.29)	1.17 (1.12-1.23)
Loss of sibling					
No	23 683 (91.4)	6522 (89.0)	1 [Reference]	1 [Reference]	1 [Reference]
Yes	2219 (8.6)	804 (11.0)	1.32 (1.21-1.44)	1.21 (1.11-1.32)	1.13 (1.02-1.27)

Abbreviation: OR, odds ratio.

^a^ Estimated using logistic regression.

^b^ Model 1 was adjusted for sex, birthplace, birth year, and conscription year.

^c^ Model 2 was additionally adjusted for parental educational level, family income, and family history of psychiatric disorders.

^d^ Model 3 was further adjusted for physical fitness and cognitive ability at conscription.

**Table 4.** Association Between Stress Resilience in Late Adolescence and Adult Psychiatric Disorders Later in Life

Outcomes	No. of cases (IR) during follow-up^a^		HR (95% CI)^b^		
	High resilience	Low resilience	Model 1^c^	Model 2^d^	Model 3^e^
Exposed to loss of either parent or sibling					
Common psychiatric disorders	9000 (11.3)	15 533 (5.1)	2.26 (2.20-2.32)	2.17 (2.11-2.23)	1.79 (1.73-1.85)
Depression	4157 (4.9)	6226 (2.0)	2.52 (2.42-2.62)	2.44 (2.34-2.54)	2.08 (1.98-2.18)
Anxiety	4511 (5.3)	6871 (2.2)	2.47 (2.37-2.56)	2.38 (2.29-2.47)	1.90 (1.81-1.99)
Substance abuse disorder	3954 (4.7)	5800 (1.8)	2.52 (2.42-2.62)	2.36 (2.26-2.46)	1.85 (1.75-1.94)
Stress-related disorders	2325 (2.7)	4046 (1.3)	2.16 (2.05-2.27)	2.03 (1.93-2.14)	1.60 (1.51-1.71)
Exposed to loss of parent					
Common psychiatric disorder	8677 (12.3)	13 844 (5.4)	2.32 (2.25-2.38)	2.21 (2.15-2.28)	1.80 (1.74-1.86)
Depression	3952 (5.2)	5585 (2.1)	2.52 (2.42-2.62)	2.43 (2.33-2.53)	2.02 (1.92-2.12)
Anxiety	4305 (5.7)	6104 (2.3)	2.50 (2.40-2.60)	2.39 (2.29-2.48)	1.89 (1.80-1.98)
Substance abuse disorder	4171 (5.6)	5396 (2.0)	2.72 (2.61-2.83)	2.54 (2.43-2.64)	1.95 (1.85-2.05)
Stress-related disorders	2291 (3.0)	3684 (1.4)	2.21 (2.10-2.33)	2.07 (1.96-2.18)	1.66 (1.56-1.77)
Exposed to loss of sibling					
Common psychiatric disorder	1371 (11.6)	2320 (5.1)	2.29 (2.14-2.46)	2.21 (2.06-2.36)	1.76 (1.61-1.92)
Depression	652 (5.2)	914 (2.0)	2.73 (2.47-3.02)	2.66 (2.40-2.95)	2.09 (1.83-2.39)
Anxiety	692 (5.5)	999 (2.1)	2.62 (2.38-2.90)	2.52 (2.29-2.79)	1.99 (1.75-2.26)
Substance abuse disorder	575 (4.6)	881 (1.9)	2.39 (2.15-2.66)	2.26 (2.03-2.51)	1.71 (1.49-1.96)
Stress-related disorders	362 (2.8)	620 (1.3)	2.20 (1.93-2.51)	2.08 (1.82-2.38)	1.59 (1.34-1.88)

Abbreviations: HR, hazard ratio; IR, incidence rate.

^a^ Number and incidence rate of psychiatric disorders per 1000 person-years.

^b^ Estimated using Cox models with time since the date of conscription as the underlying time scale.

^c^ Model 1 was adjusted for sex, birthplace, birth year, and conscription year.

^d^ Model 2 was additionally adjusted for parental educational level, family income, and family history of psychiatric disorders.

^e^ Model 3 was further adjusted for physical fitness and cognitive ability at conscription.

### The Mediating Role of Stress Resilience in Associations Between Childhood Bereavement and Risk of Psychiatric Disorders

There was a statistically significant mediating effect of stress resilience on the association between childhood bereavement and psychiatric disorder risk later in life (Table 5), with the estimated proportion of the total effect mediated by stress resilience ranging from 10.6% for substance abuse disorder to 19.4% for anxiety due to loss of either a parent or sibling and from 15.6% for depression to 21.7% for anxiety due to loss of a parent. For loss of a sibling, the estimated proportion mediated by stress resilience was 18.4% for any common psychiatric disorder and 6.2% for stress-related disorders, respectively.

**Table 5.** Mediation Analyses of Stress Resilience on the Association Between Childhood Bereavement and Adult Psychiatric Disorders^a^

Effect	HR (95% CI)^b^				
	Common psychiatric disorder	Depression	Anxiety	Substance abuse disorder	Stress-related disorder
Exposed to loss of either parent or sibling					
Natural direct effect	1.21 (1.16-1.26)	1.21 (1.14-1.29)	1.14 (1.08-1.21)	1.31 (1.23-1.39)	1.17 (1.08-1.27)
Natural indirect effect	1.03 (1.02-1.03)	1.03 (1.03-1.04)	1.03 (1.02-1.04)	1.03 (1.02-1.03)	1.02 (1.02-1.03)
Total effect	1.24 (1.19-1.29)	1.25 (1.18-1.33)	1.18 (1.11-1.25)	1.35 (1.27-1.43)	1.19 (1.10-1.29)
Proportion mediated, %	13.2^c^	16.6^c^	19.4^c^	10.6^c^	12.6^c^
Exposed to loss of parent					
Natural direct effect	1.13 (1.08-1.18)	1.17 (1.10-1.25)	1.10 (1.03-1.17)	1.14 (1.07-1.22)	1.07 (0.99-1.17)
Natural indirect effect	1.02 (1.02-1.03)	1.03 (1.02-1.03)	1.02 (1.02-1.03)	1.03 (1.02-1.03)	1.02 (1.01-1.02)
Total effect	1.16 (1.11-1.21)	1.20 (1.13-1.29)	1.12 (1.05-1.20)	1.17 (1.10-1.25)	1.09 (1.01-1.19)
Proportion mediated, %	15.9^c^	15.6^c^	21.7^c^	17.0^c^	20.8^c^
Exposed to loss of sibling					
Natural direct effect	1.09 (0.98-1.21)	1.06 (0.90-1.24)	1.04 (0.89-1.22)	1.02 (0.86-1.21)	1.26 (1.04-1.53)
Natural indirect effect	1.02 (1.00-1.03)	1.02 (1.01-1.04)	1.02 (1.01-1.04)	1.02 (1.00-1.03)	1.01 (1.00-1.02)
Total effect	1.10 (0.99-1.23)	1.08 (0.92-1.27)	1.07 (0.91-1.25)	1.04 (0.87-1.23)	1.28 (1.05-1.55)
Proportion mediated, %	18.4^d^	31.0	34.9	48.7	6.2^d^

Abbreviation: HR, hazard ratio.

^a^ The association between childhood bereavement and psychiatric disorders was estimated using Cox models. Stress resilience was dichotomized as low (1-3) or high (4-9), and mediation effect was estimated using logistic regression.

^b^ Estimates were adjusted for sex, birth year, birthplace, conscription year, parental educational level, family income, family history of psychiatric disorders, and physical fitness and cognitive ability at conscription.

^c^ *P* < .001 for the estimated proportion of mediation.

^d^ *P* < .05 for the estimated proportion of mediation.

## Discussion

In this study, we used nationwide Swedish register data with a follow-up of up to 34 years to examine the role of stress resilience in associations between childhood bereavement and risk of psychiatric disorders in adulthood. Our findings show stress resilience in late adolescence partially mediates the association between exposure to childhood bereavement and adult psychiatric disorders.

To our knowledge, our study is the first to comprehensively assess the role of stress resilience in the association between bereavement and psychiatric disorders using prospective data. Yet, our results align with previous findings highlighting the increased risk of psychiatric disorders and mental disorder symptoms after childhood bereavement.^2,3,4,6,9,10^ As such, our findings converge with existing evidence suggesting that childhood bereavement represents an important risk factor for adverse mental health outcomes later in life.

While associations between childhood bereavement and increased risk of depression have been documented for more than 60 years,^37^ we extend previous findings by showing that childhood bereavement, in particular parental loss, is associated with increased risk of common psychiatric diagnoses, including depression, anxiety, substance use, and stress-related disorders. We assessed psychiatric disorder risk over decades, which, to our knowledge, is the longest follow-up period of any study examining the link between childhood bereavement and psychiatric disorders to date.

We also found that stress resilience had a stronger association with risk of psychiatric disorders compared with childhood bereavement. While this could be explained (partially or fully) by temporal effects (ie, resilience was assessed closer to the onset of psychiatric disorders), it is also suggestive of the relevance of resilience for psychiatric disorder risk in adulthood. As most individuals experience stressful life events over their lifetime, the impact of these on adverse mental health outcomes may partly be shaped by resilience factors.^13^

Comparing our findings with previous research is challenging given the different approaches used to conceptualise and measure stress resilience.^16^ Our key finding that stress resilience partially mediated the association of childhood bereavement with psychiatric disorder risk aligns with a recent meta-analysis that found that trait resilience partly mediated the association between childhood stressors and depressive symptoms in adulthood, although this study was based solely on cross-sectional data.^21^ Using longitudinal and prospectively collected data, our findings suggest that altered stress resilience may be 1 mechanism for how childhood bereavement impacts risk of psychiatric disorders later in life.

### Strengths and Limitations

There are several strengths of our study. We used a nationwide study population with linkages across multiple population-based registers, allowing for detailed analyses by subgroups and adjustment for covariates. This addresses a key limitation of existing studies of resilience, adversity, and mental health, namely the use of small and nonrepresentative samples.^20^ Furthermore, data on bereavement exposure and psychiatric disorders were independently and prospectively collected, reducing risk of bias due to selection and differential report. As previously noted, our follow-up period included several decades.

Our findings should be interpreted considering several important limitations. First, information on the assessment procedure of stress resilience, while conducted by trained psychologists, is not publicly available. Thus, we lack knowledge regarding the specific aspects of resilience assessed. Second, we cannot exclude the possibility of unmeasured confounding (eg, by genetic factors) in observed associations. Recent studies have identified genetic and gene-environment interaction effects involved in the relationship between stressful life experiences and mental health outcomes.^38,39^ Causal mediation analyses rely on several assumptions, including the absence of confounding in the relationships between the exposure and outcome, the exposure and mediator, the mediator and outcome, and no mediator-outcome confounder caused by the exposure. It is difficult to examine whether any of these assumptions has been violated in the present study, as in any other observational study. As a result, we cannot rule out the risk of potential bias in the causal mediation analyses and must remain cautious when interpretating these results. Further triangulation across study designs with orthogonal sources of bias is therefore warranted. Third, we used register-based data from Sweden and did not assess the association between bereavement and less severe psychiatric outcomes or less commonly occurring psychiatric disorders. Fourth, resilience was only measured once, limiting insights into how resilience could change during the lifespan. Fifth, we excluded participants diagnosed with a psychiatric disorder before conscription from the study, which could have introduced selection bias,^40^ as preconscription diagnoses may influence eligibility for conscription and, consequently, study inclusion. The sensitivity analysis including participants with a diagnosis of psychiatric disorders before conscription showed similar results as the ones obtained in the main analysis, demonstrating the limited impact of such bias. However, as individuals with a psychiatric disorder diagnosed in childhood are believably less likely to be conscripted than others, there is regardless a concern of selection bias. Additionally, the generalizability of our findings beyond the Swedish context is unclear. Furthermore, as 98.5% of the study participants were male, the extent to which the observed associations may apply for females is not clear. Relatedly, whether there is an interaction between sex of the offspring and sex of the deceased parent or sibling remains to be investigated, although we did not find the study results to greatly vary between loss of a mother or a father.

## Conclusions

In this cohort study of a nationwide Swedish sample, we found that childhood bereavement was associated with increased risk of common psychiatric disorders in adulthood, and that stress resilience measured in late adolescence partly mediated the association of childhood bereavement with risk of psychiatric disorders later in life. Our findings suggest that altered stress resilience is one mechanism through which loss of a parent or sibling in childhood is associated with risk of psychiatric disorders in adulthood.

## Notes

Supplement 1. **eFigure.** Study Design **eTable 1. **Psychiatric Disorders Assessed in the Study **eTable 2. **HRs With 95% CIs for Risk of Psychiatric Disorders Among Individuals Exposed to Childhood Bereavement Compared With Matched Unexposed Individuals, Stratified Analysis by Characteristics **eTable 3. **Crude IRs and HRs With 95% CIs for adult psychiatric disorders among individuals Exposed to Childhood Bereavement Compared With Matched Unexposed Individuals, by Time of Follow-Up (Before Conscription and After Conscription)

Supplement 2.
Data Sharing Statement
