# Supplementary material for: Stress Resilience and Risk of Psychiatric Disorders After Childhood Bereavement
Source: JAMA Netw Open. 2025 Jul 9;8(7):e2519706. doi: 10.1001/jamanetworkopen.2025.19706 (PMC12242700; doi:10.1001/jamanetworkopen.2025.19706)
Supplement: Supplement 1. — eFigure. Study Design eTable 1. Psychiatric Disorders Assessed in the Study eTable 2. HRs With 95% CIs for Risk of Psychiatric Disorders Among Individuals Exposed to Childhood Bereavement Compared With Matched Unexposed Individuals, Stratified Analysis by Characteristics eTable 3. Crude IRs and HRs With 95% CIs for adult psychiatric disorders among individuals Exposed to Childhood Bereavement Compared With Matched Unexposed Individuals, by Time of Follow-Up (Before Conscription and After Conscription) [file jamanetwopen-e2519706-s001.pdf]

Supplemental Online Content

Bjørndal LD, Chen Y, Lu D, et al. Stress resilience and risk of psychiatric disorders after childhood bereavement. *JAMA Netw Open*. 2025;8(7):e2519706. doi:10.1001/jamanetworkopen.2025.19706

eFigure. Study Design
eTable 1. Psychiatric Disorders Assessed in the Study
eTable 2. HRs With 95% CIs for Risk of Psychiatric Disorders Among Individuals Exposed to Childhood Bereavement Compared With Matched Unexposed Individuals, Stratified Analysis by Characteristics
eTable 3. Crude IRs and HRs With 95% CIs for adult psychiatric disorders among individuals Exposed to Childhood Bereavement Compared With Matched Unexposed Individuals, by Time of Follow-Up (Before Conscription and After Conscription)

This supplemental material has been provided by the authors to give readers additional information about their work.

© 2025 Bjørndal LD et al. *JAMA Network Open*.

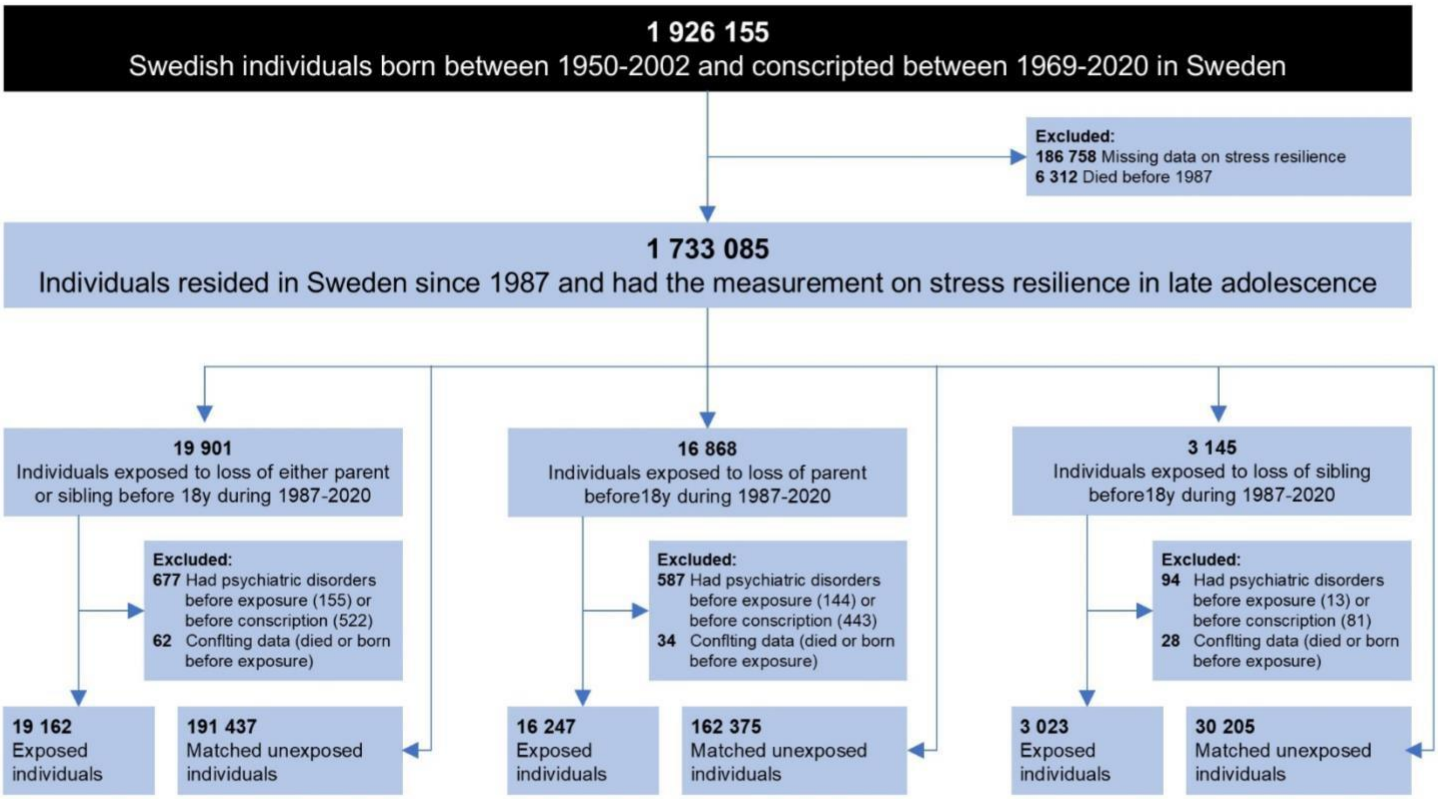

eFigure. Study Design
Data were cross-linked between the Swedish Military Conscription Register, National Patient Register, Causes of Death Register, and unexposed individuals in the population-matched cohorts (1:10 individually matched to the exposed individual by sex, birth year, and birthplace) were randomly selected from the study population (N=1 733 085), using the incidence density sampling method. Unexposed individuals were free of the interested exposure (i.e., bereavement of a specific type) and free of psychiatric disorders before index date (i.e., date of bereavement for the exposed individual and their matched unexposed individuals).

© 2025 Bjørndal LD et al. *JAMA Network Open*.

eTable 1. Psychiatric Disorders Assessed in the Study

Psychiatric disorder	ICD codes
Common psychiatric disorder	ICD-9: includes ICD-9 codes for depression, anxiety, substance abuse disorder, and stress-related disorders as shown below
	ICD-10: includes ICD-10 codes for depression, anxiety, substance abuse disorder, and stress-related disorders as shown below
Depression	ICD-9: 296B, 296D, 298A, 300E, 311
	ICD-10: F32, F33
Anxiety	ICD-9: 300A, 300C
	ICD-10: F40, F41
Substance abuse disorder	ICD-9: 291, 292, 303-305
	ICD-10: F10-F19
Stress-related disorder	ICD-9: 308-309
	ICD-10: F43

© 2025 Bjørndal LD et al. *JAMA Network Open*.

eTable 2. HRs With 95% CIs for Risk of Psychiatric Disorders Among Individuals Exposed to Childhood Bereavement Compared With Matched Unexposed Individuals, Stratified Analysis by Characteristics

**Characteristics**	**Loss of either parent or sibling**		**Loss of parent**		**Loss of sibling**	
	**HR (95% CI)** [a]	$P_{interaction}$ [b]	**HR (95% CI)** [a]	$P_{interaction}$ [b]	**HR (95% CI)** [a]	$P_{interaction}$ [b]
Sex						
Male	1.21 (1.16-1.27)	0.055	1.14 (1.09-1.20)	0.21	1.11 (1.00-1.24)	0.87
Female	1.02 (0.80-1.29)		1.07 (0.82-1.40)		1.20 (0.71-2.03)	
Age at index date, years						
<6	1.11 (0.96-1.27)	0.45	1.16 (0.98-1.37)	0.87	1.14 (0.92-1.43)	0.92
6-12	1.25 (1.16-1.36)		1.17 (1.08-1.28)		1.14 (0.93-1.39)	
>12-18	1.20 (1.14-1.27)		1.13 (1.07-1.19)		1.08 (0.92-1.27)	
Attained age, years						
<30	1.04 (0.94-1.15)	0.59	1.06 (0.96-1.18)	0.94	0.90 (0.70-1.15)	0.53
30-40	1.17 (1.05-1.29)		1.09 (0.98-1.22)		1.15 (0.88-1.51)	
>40	1.03 (0.88-1.21)		1.11 (0.95-1.31)		0.88 (0.55-1.39)	
Annual family income level						
Lowest 20%	1.57 (0.57-4.31)	0.70	0.75 (0.26-2.19)	0.76	NA	0.26
Middle	1.23 (1.17-1.29)		1.14 (1.08-1.20)		1.15 (1.00-1.31)	
Top 20%	1.14 (1.02-1.28)		1.22 (1.07-1.39)		1.09 (0.86-1.38)	
Family history of psychiatric disorders						
No	1.21 (1.15-1.27)	0.70	1.15 (1.09-1.21)	0.40	1.10 (0.98-1.24)	0.76
Yes	1.30 (1.12-1.50)		1.11 (0.97-1.27)		1.22 (0.71-2.09)	
Year of conscription						
1969-2006	1.21 (1.16-1.27)	0.07	1.14 (1.09-1.20)	0.40	1.11 (0.99-1.25)	0.95
2007-2020	1.18 (1.02-1.36)		1.06 (0.91-1.24)		1.19 (0.88-1.62)	
Physical fitness						
High (5-9)	1.19 (1.13-1.25)	0.45	1.13 (1.07-1.18)	0.79	1.09 (0.96-1.23)	0.09
Low (0-4)	2.86 (1.08-7.57)		1.06 (0.49-2.30)		1.84 (0.19-17.82)	
Cognitive ability						
High (4-9)	1.21 (1.15-1.27)	0.72	1.14 (1.08-1.21)	0.93	1.08 (0.94-1.23)	0.48
Low (1-3)	1.24 (1.10-1.38)		1.15 (1.03-1.30)		1.10 (0.81-1.49)	
Sex of deceased parent or sibling						
Male	1.22 (1.16-1.28)	0.53	1.17 (1.11-1.24)	0.28	1.11 (0.97-1.27)	0.99
Female	1.18 (1.10-1.27)		1.07 (0.99-1.16)		1.11 (0.94-1.33)	

HR, hazard ratio; CI, confidence interval. NA, not available, due to small sample size in this stratum.
[a] Estimates were calculated from Cox models, adjusted for sex, birth year, and birthplace, conscription year, parental educational level, family income, family history of psychiatric disorders, and physical fitness and cognitive ability at conscription, if applicable.
[b] *P* value from two-sided likelihood ratio test for heterogeneity of HRs across subgroups.

© 2025 Bjørndal LD et al. *JAMA Network Open*.

eTable 3. Crude IRs and HRs With 95% CIs for adult psychiatric disorders among individuals Exposed to Childhood Bereavement Compared With Matched Unexposed Individuals, by Time of Follow-Up (Before Conscription and After Conscription)

	**Before conscription**			**After conscription**		
**Outcomes**	**No of cases (IR [a]) in exposed individuals**	**No of cases (IR [a]) in unexposed individuals**	**HR (95% CI) [b]**	**No of cases (IR [a]) in exposed individuals**	**No of cases (IR [a]) in unexposed individuals**	**HR (95% CI) [b]**
**Exposed to loss of either parent or sibling**						
Common psychiatric disorders	422 (3.5)	2398 (2.0)	1.37 (1.22-1.55)	3077 (9.0)	21006 (6.0)	1.23 (1.18-1.28)
Depression	89 (0.7)	489 (0.4)	1.35 (1.01-1.82)	1396 (3.8)	8920 (2.4)	1.24 (1.17-1.32)
Anxiety	74 (0.6)	422 (0.4)	1.17 (0.85-1.63)	1489 (4.1)	9907 (2.7)	1.20 (1.13-1.27)
Substance abuse disorder	252 (2.1)	1584 (1.3)	1.20 (1.03-1.40)	1425 (4.0)	8169 (2.2)	1.34 (1.25-1.42)
Stress-related disorders	86 (0.7)	222 (0.2)	3.83 (2.77-5.29)	881 (2.4)	5632 (1.5)	1.22 (1.13-1.32)
**Exposed to loss of parent**						
Common psychiatric disorders	363 (3.7)	2198 (2.3)	1.35 (1.18-1.53)	2712 (9.3)	19767 (6.6)	1.12 (1.07-1.17)
Depression	71 (0.7)	433 (0.5)	1.31 (0.95-1.79)	1239 (4.0)	8375 (2.7)	1.19 (1.12-1.27)
Anxiety	64 (0.7)	429 (0.4)	1.14 (0.82-1.58)	1316 (4.2)	9373 (3.0)	1.07 (1.00-1.14)
Substance abuse disorder	219 (2.2)	1430 (1.5)	1.20 (1.02-1.41)	1280 (4.2)	8294 (2.7)	1.13 (1.06-1.21)
Stress-related disorders	72 (0.7)	214 (0.2)	3.25 (2.32-4.57)	761 (2.4)	5555 (1.8)	1.04 (0.96-1.13)
**Exposed to loss of sibling**						
Common psychiatric disorders	61 (2.3)	425 (1.6)	1.38 (1.03-1.85)	391 (7.6)	3368 (6.4)	1.07 (0.96-1.19)
Depression	18 (0.7)	98 (0.4)	1.29 (0.67-2.48)	172 (3.2)	1444 (2.6)	1.08 (0.92-1.28)
Anxiety	10 (0.4)	83 (0.3)	0.86 (0.35-2.09)	189 (3.5)	1619 (3.0)	1.05 (0.90-1.23)
Substance abuse disorder	34 (1.3)	260 (1.0)	1.26 (0.86-1.85)	150 (2.8)	1391 (2.6)	0.95 (0.80-1.13)
Stress-related disorders	15 (0.6)	43 (0.2)	4.16 (1.96-8.83)	127 (2.3)	889 (1.6)	1.25 (1.03-1.52)

IR, incidence rate; HR, hazard ratio; CI, confidence interval.
[a] Incidence rate of psychiatric disorders per 1000 person-years.
[b] Estimates were calculated from Cox models with time since the index date as the underlying time scale, and adjusted for matching identifier (sex, birth year, and birthplace), conscription year, parental educational level, family income, family history of psychiatric disorders, and physical fitness and cognitive ability at conscription.

© 2025 Bjørndal LD et al. *JAMA Network Open*.
