# Supplementary material for: Stress Resilience and Risk of Psychiatric Disorders After Childhood Bereavement
Source: JAMA Netw Open. 2025 Jul 9;8(7):e2519706. doi: 10.1001/jamanetworkopen.2025.19706 (PMC12242700; doi:10.1001/jamanetworkopen.2025.19706)
Supplement: Supplement 2. — Data Sharing Statement [file jamanetwopen-e2519706-s002.pdf]

Data Sharing Statement

Bjørndal. Stress Resilience and Risk of Psychiatric Disorders After Childhood Bereavement. *JAMA Netw Open*. Published July 09, 2025. doi:10.1001/jamanetworkopen.2025.19706

Data
Data available: No
